# Medium-term monitoring reveals effects of El Niño Southern Oscillation climate variability on local salinity and faunal dynamics on a restored oyster reef

**DOI:** 10.1371/journal.pone.0255931

**Published:** 2021-08-16

**Authors:** Jennifer Beseres Pollack, Terence A. Palmer, Abby E. Williams

**Affiliations:** 1 Harte Research Institute, Texas A&M University-Corpus Christi, Corpus Christi, Texas, United States of America; 2 Department of Life Sciences, Texas A&M University-Corpus Christi, Corpus Christi, Texas, United States of America; Universidad de Antioquia, COLOMBIA

## Abstract

Human activities and regional-scale climate variability drive changes in the ecology of coastal and marine ecosystems. Ecological restoration has emerged as a best-management practice to combat habitat degradation and restore lost ecological functions. However, relatively short project monitoring timeframes have limited our understanding of the effects of interannual climate cycles on water quality and restoration dynamics. We collected measurements on a 23-ha oyster reef constructed in the Gulf of Mexico to determine the relationship between El Niño Southern Oscillation (ENSO)-driven climate variability and local salinity patterns, and to evaluate the effects of this climate variability and salinity on oyster population dynamics and faunal community composition over a medium-term (five-year) timeframe. The role of ENSO-driven climate variability on local salinity patterns (primarily from changes in precipitation and evaporation) and faunal dynamics was investigated using the Oceanic Niño Index (ONI). Salinity was negatively correlated with ONI with an approximately 4-month lag. Higher ONI values (El Niño periods) were followed by reductions in salinity, increases in oyster recruitment and density, and reductions in resident motile fauna density and species richness. Lower ONI values (La Niña periods) had higher and less variable salinities, and higher areal coverage of restoration substrates by large oysters. ENSO-driven salinity reductions in the second year after reef construction coincided with a shift in resident motile faunal community composition that was maintained despite a second strong salinity reduction in year 5. Our results indicate that it is important to expand the typical monitoring timeframes to at least five years so that resource managers and restoration practitioners can better understand how both short-term environmental variability and longer-term climate cycles can affect the outcomes of restoration actions.

## Introduction

Substantial degradation of estuarine and coastal ecosystems is a global problem that is heavily influenced by multiple human activities [1]. Many degraded ecosystems have lost vast quantities of key habitats and now have reduced biodiversity and resilience [2]. Ecological restoration has emerged as a best management practice to combat habitat loss in estuarine and coastal ecosystems [3], with the primary goal of replacing lost ecosystem functions and services [4]. Coastal ecosystems are more expensive to restore than freshwater and terrestrial systems, and the success of restoration projects are not always proportional to their costs [5]. This cost inefficiency compounds the need for information that can be used to improve restoration practices.

In the U.S. Gulf of Mexico (GoM), El Niño Southern Oscillation (ENSO) influences local and regional weather patterns [6, 7]. ENSO cycles occur irregularly every 2–7 years (average every 3–4 years [8], with precipitation and freshwater delivery to the GoM increasing during the El Niño phase and decreasing with La Niña [9–11]. These differences in regional precipitation have been linked to variability in estuarine salinities [11–13] and effects on ecological patterns and processes [13–16]. ENSO phases can be classified using the Oceanic Niño Index (ONI) [17], which uses 3 month running means of sea surface temperature (SST) anomalies in the Niño 3.4 region (5° N to 5° S, 120° W to 170° W) [18], and has become the index of choice for ENSO monitoring and prediction [19, 20]. ENSO events are classified as a minimum of five consecutive months with ONI > +0.5°C (El Niño) or ONI< -0.5°C (La Niña) [17].

Successful restoration planning and implementation require an understanding of ecological responses to climate variability [21]. Because restoration monitoring typically occurs over short-term (e.g. 1–2 years [5, 22]) time frames dictated by funding, it is often not possible to assess the effects of interannual climate variability on restoration dynamics. To improve the efficacy of restoration to recover coastal ecosystems and their associated services, it is critical to incorporate sustained measurements over ecologically relevant time frames for recovery [23, 24]. Despite the multitude of restoration projects occurring, many have had limited or no monitoring [25–27], and comprehensive evaluations are rare [28, 29]. Minimum periods for restoration monitoring should incorporate the periodicity of climate cycles that affect ecological dynamics. Incorporating longer assessment timeframes can improve our ability to validate short-term restoration findings and to understand and manage responses to large-scale estuarine variability [30, 31], and are a key step toward producing positive returns on restoration investments [32].

Eastern oyster *Crassostrea virginica* populations have experienced severe declines over the past two centuries [33], and the majority of U.S. restoration efforts have occurred in the GoM [32]. Assessing restored habitats over medium-term time frames (e.g. 4–6 years post-construction) [34] is important for planning and implementing effective restoration and understanding the effects of large-scale climate variability. Salinity can impact survival, growth, and reproduction of oysters in the GoM by altering filtration and respiration rates [35], disease dynamics [36, 37] and predation [38], and ENSO-driven salinity variability has been linked to effects on oyster growth and fitness as well as disease [39–41].

In this study, we examined whether environmental and climate variables influenced local oyster reef development and faunal habitat provision on a constructed oyster reef in the Gulf of Mexico. Monitoring of the reef occurred quarterly (seasonally) for five years following reef construction, encompassing 1.3 El Niño and 2 La Niña periods. The objectives of this study were to determine the relationship between ENSO-driven climate variability and local salinity patterns, and the effects of this climate variability and salinity on oyster population dynamics and faunal community composition over a medium-term (five-year) timeframe. Scaling restoration monitoring timeframes to allow for adequate assessment of climate-driven effects on restoration dynamics will improve our ability to predict and manage for long-term success of restoration efforts.

## Methods

### Study site

**Fig 1:**
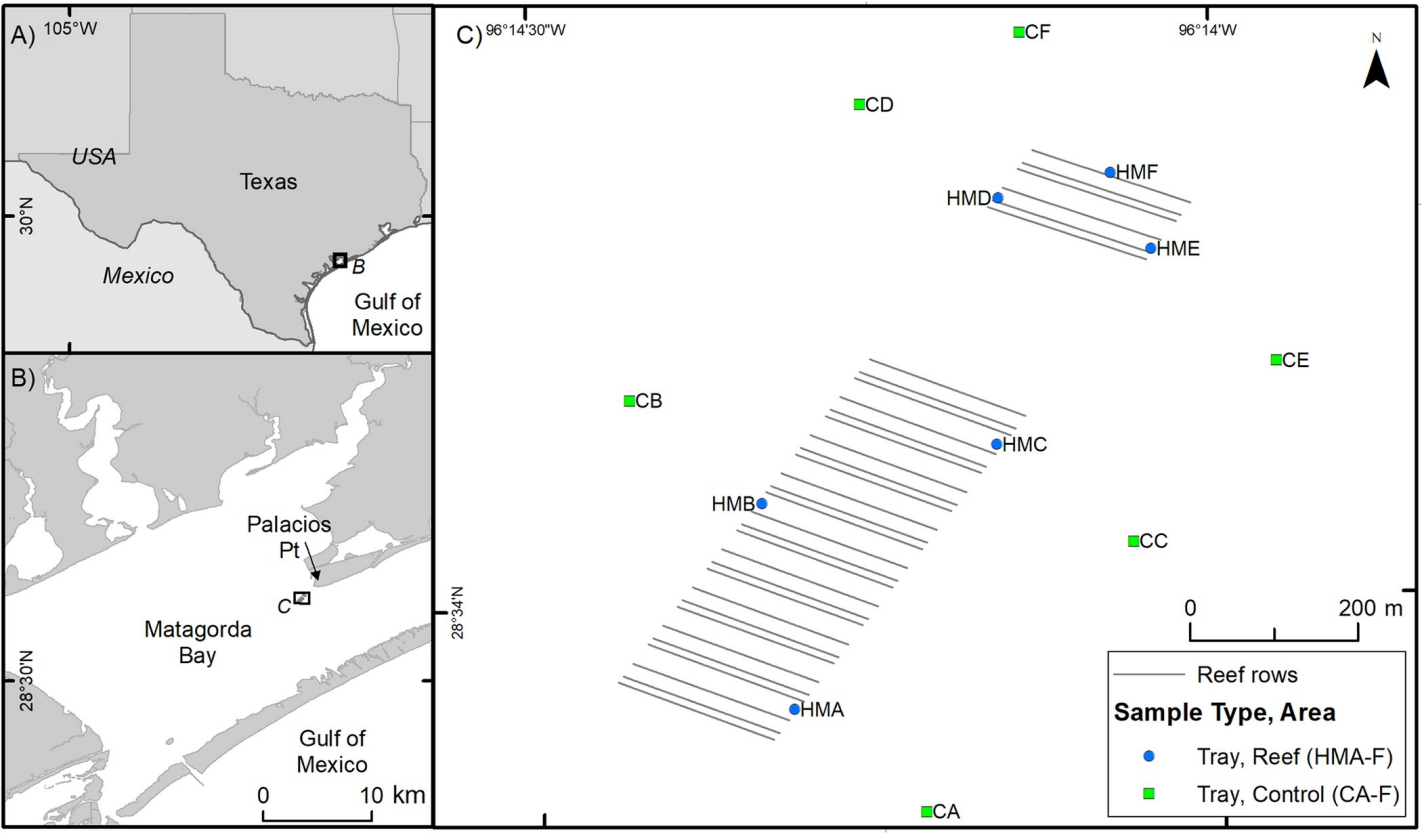
Study area and sampling sites at Half Moon Reef, Matagorda Bay, Texas, USA. The constructed reef was designed to maximize vertical relief and reef edge, resist wave damage, and minimize sediment accumulation. The HM and C prefixes of site names refer to Half Moon Reef (constructed reef) and Control sites, respectively.

Half Moon Reef is a historic 200 ha *Crassostrea virginica* subtidal oyster reef in Matagorda Bay, in the western Gulf of Mexico. Oyster populations on the reef collapsed in the early 1900s due to intensive harvest activities and loss of reef structure [42] and were rendered functionally extinct in the 1920s [43]. In 2011, surveys determined that the former reef area was composed of primarily mud and shell hash and lacked any complex structure [44]. In March-April 2014, 23 ha of reef were constructed by The Nature Conservancy using limestone and concrete cobble, comprising a series of 98 reef rows of dimensions 189 m long x 18 m wide x 1–1.5 m high (Fig 1). The reef was designed to maximize vertical relief and amount of reef edge, be resistant to wave damage, and have an orientation that minimizes sediment deposition and accumulation [45].

Monitoring of the reef was carried out quarterly (four times yr^-1^) from April 2014 to January 2019 at six fixed sampling sites on the constructed reef (HMA-HMF) and six control sites (CA-CF) located 150 m away. No natural reefs exist within the vicinity (~10 km) of the constructed reef. Monitoring of earlier years of this restoration project were previously reported by [44] and [46]. All twelve sites were sampled during each sampling event until May 2017 and ten sites were sampled thereafter. We discontinued sampling of HMF and CF in this project after May 2017 due to the loss of sampling trays.

### Environmental and climate variability

Discrete measurements of temperature (°C), dissolved oxygen (mg l^-1^), and salinity were made at the surface and the bottom of the water column at multiple sampling sites on each sampling date using a YSI data sonde (no replicates at each site). Discrete salinity data was more representative of the salinity at Half Moon Reef than data from the closest continuous (daily) monitoring station (Site NCM4) [47] because Half Moon Reef salinities were on average 3 units higher and the Reef does not receive the same frequency and magnitude of low salinity pulses as the continuous monitoring station [46]. The potential role of climate variability on local salinity patterns and faunal dynamics was investigated using ONI data based on the 1986–2015 base period [17]. The relationship between ONI and salinity was determined by calculating Spearman-rank correlations between simultaneous and temporally-lagged ONI data (1- to 6-month lags) with mean bottom-water (≥1 m) salinity, water temperature, dissolved oxygen concentration and pH. Temporal lags account for any delay in changes in salinity after a change within the ONI climate cycle.

### Reef development

Divers retrieved at least two representative pieces of substrate (30 to 50 cm diameter) from each constructed reef site in each sampling event (quarterly frequency, starting in July 2014) to characterize and quantify the oyster population. A piece of flexible mesh (≥ 60 cm^2^) with 2.5 cm length × 2 cm width grid size was overlaid on two areas of each substrate piece to estimate percent areal cover. The area measured was selected haphazardly from the any area of the substrate pieces that were directly exposed to the water column (i.e., areas that were sitting on the seabed or against other rocks were avoided). All live oysters were enumerated and measured for shell height. The pieces were returned after measurements were taken. Up to 20 oysters (> 25 mm) per sampling event were held on ice and brought back to the laboratory to determine presence and severity of the protozoan parasite *Perkinsus marinus* using the culture method of [48]. *Perkinsus marinus* causes severe oyster mortalities in the Gulf of Mexico and disease dynamics can be strongly influenced by ENSO cycles; La Niña conditions create higher water temperatures and salinities, which are favorable for *P*. *marinus* susceptibility and disease transmission [40]. Infection intensity (II) was scored using the 6-point Mackin scale (uninfected (0)—heavily infected (5)) adapted from [49] by [50]. The prevalence of oysters infected with *P*. *marinus* was calculated by dividing the number of infected oysters by the number of oysters sampled. Weighted prevalence, a measure of the relative severity of *P*. *marinus* infection in a population, was calculated by multiplying the mean infection intensity of all oysters at a site on a certain date the by prevalence at the same site and date.

Spearman correlations were calculated to determine the relationship between environmental and climate (ONI) variables with oyster density and areal coverage of reef construction substrates by oysters. Temporal summaries of mean oyster densities and disease variables were made using annual means, starting from when the reef was constructed (sampling year, March to February). Oyster size-frequency distributions were made by binning all oysters within each sampling year. Univariate analyses and data management were performed using SAS 9.4 [51].

### Habitat provision

Two sampling trays of dimensions 46 x 61 cm (0.28 m^2^) were deployed in April 2014 at each sampling site and were filled with concrete cobble (reef construction substrate) at the constructed reef sites and soft sediment at the control sites. The purpose of the trays filled with cobbles was to enable a quantifiable area of the reef to be sampled for resident motile fauna. Placing two sampling trays at each site allowed for each tray to be sampled only once every six months, to minimize the disturbance of removing most motile fauna. to assess differences in habitat provision. Resident motile fauna (> 1mm) were collected quarterly from a single sampling tray at each site starting in July 2014 by divers using a suction sampler (Honda 160cc semi-trash pump with 5.1 cm ports) [44]. Trays remained in position throughout the study period due to their large size and weight (> 20 kg). Fauna were fixed in 10% buffered formalin in the field and brought back to the laboratory where they were sorted, counted, and identified to the lowest taxonomic level possible. Dry weights of organisms were obtained by placing samples in an oven at 55°C for at least 24 hours. Mollusk shells were removed prior to biomass measurements using 1 mol L^-1^ HCl.

Spearman correlations were calculated to determine the relationship between environmental and climate (ONI) variables with faunal density, biomass, and species richness. Non-metric multidimensional scaling analysis (nMDS) [52] with a Bray-Curtis similarity matrix was overlaid with cluster analysis (group average method) to describe differences in motile fauna assemblages. Similarity profile analysis (SIMPROF) was used to test for significance within clusters. Density data were log_e_(x+1) transformed and biomass data were fourth root transformed. Similarity percentage analyses (SIMPER) were used to describe taxa that were characteristic of, and different among, treatments and dates. The BIO-ENV procedure was used to correlate community composition with combinations of environmental and climate variables using Spearman-rank correlations [53]. Temporal summaries for faunal community variables in this study were made using annual mean community variables starting from when the reef was constructed (March to February). All multivariate analyses were conducted using PRIMER 7 [54].

This study was carried out in strict accordance with recommendations in the Public Health Service Policy on Humane Care and Use of Laboratory Animals from the National Institutes of Health. The protocol was approved by the Institutional Animal Care and Use Committee of Texas A&M University-Corpus Christi (Protocol Numbers: 08–14, 09–14, 10–17). Field collection of organisms was conducted in accordance with Texas Parks and Wildlife Department Scientific Permit Number SPR-0911-344.

## Results

### Environmental and climate variability

**Fig 2:**
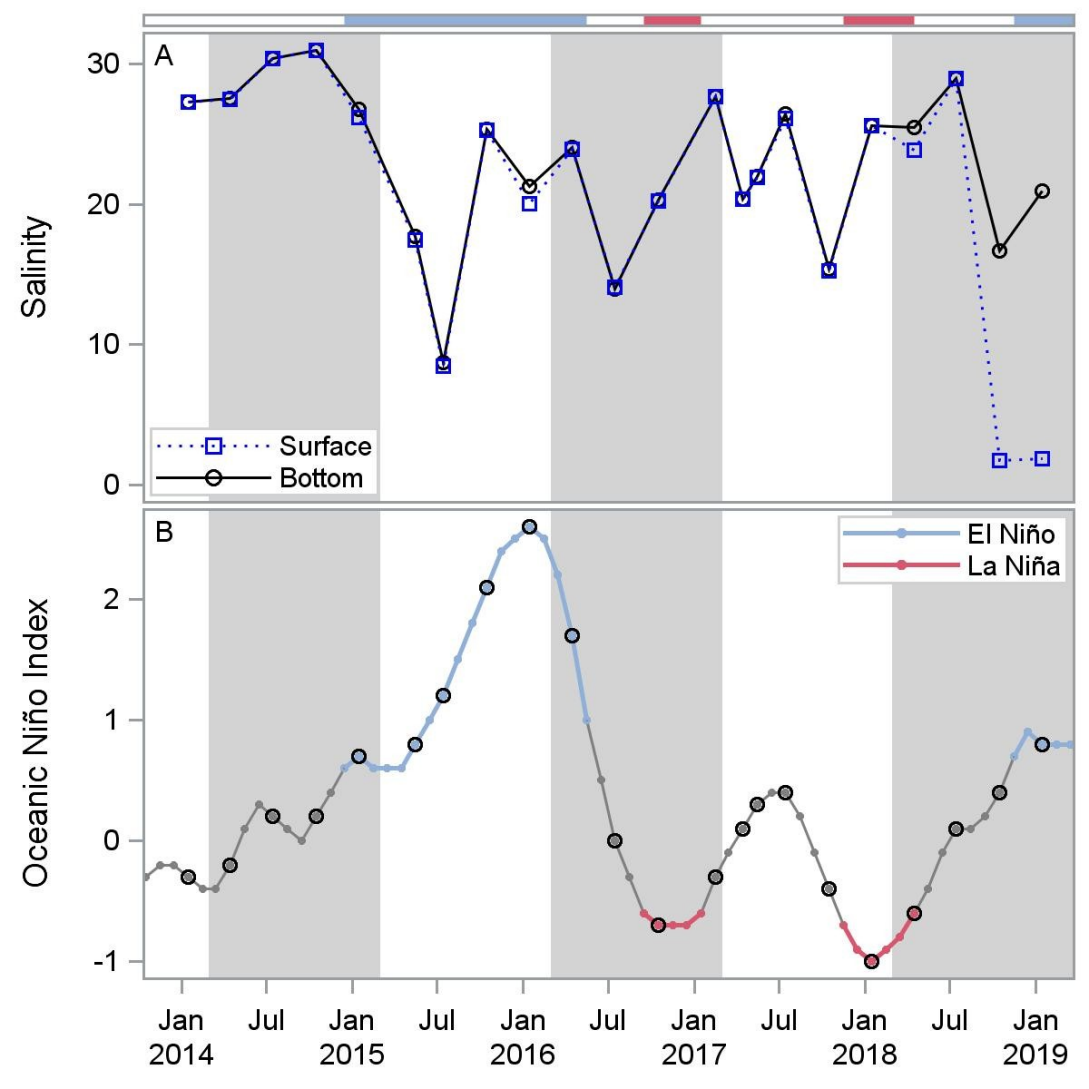
Mean salinity at Half Moon Reef at the surface (< 1 m depth) and bottom (> 1 m depth, measured quarterly; A) and Oceanic Niño Index (ONI; B), measured monthly, from January 2014-January 2019. Large points on ONI graph indicate sampling dates. Colors on ONI graph and horizontal bar indicate ENSO state: light blue = El Niño, red = La Niña. ONI data are from [17].

Salinities averaged (mean ± standard error) 21.3 ± 5.6 and fluctuated widely over the course of the study, ranging from 1.8 at the surface in October 2018 to 30.9 in October 2014 (Fig 2). Two notable reductions in salinity occurred: one between the first and second year after reef construction throughout the whole water column (from 26.7 in January 2015 to 8.7 in July 2015) and another in the fifth year after reef construction in surface waters (from 28.9 in July 2018 to 1.8 in October 2018), both caused by heavy rains over the bay and watershed. Vertical stratification in salinity in October 2018 and January 2019 is likely the result of sustained high inflow volumes from the Colorado River, the primary freshwater source to Matagorda Bay, and limited vertical mixing. Temperatures averaged 23.7 ± 6.5°C and displayed expected seasonal variation, with mean values ranging from 9.6°C in January 2015 to 31.5°C in July 2017 (S1 Fig). Differences in interannual temperatures are attributed to differences in dates sampled rather than true interannual variation. Dissolved oxygen concentrations averaged 7.4 ± 1.3 mg l^-1^ and were inversely related to temperature, ranging from 5.7 mg l^-1^ in July 2014 to 10.9 mg l^-1^ in January 2018. pH averaged 8.1 ± 0.1 and ranged from 7.8 at bottom waters in January 2019 to 8.7 in July 2014. pH was similar at the top and bottom on all dates aside from in January 2019 when there was vertical salinity stratification.

El Niño conditions were present in the first and second years after reef construction (mid-2014 to mid-2016) and transitioned to La Niña conditions in the third and fourth years after reef construction (mid-2016 to mid-2018); El Niño conditions returned during the fifth year (mid-2018 to 2019). Salinity was negatively correlated with ONI with 2-month (r = -0.43, p ≤ 0.04), 3- month (r = -0.48, p ≤ 0.02), 4-month (r = -0.51, p ≤ 0.02), and 5-month (r = -0.47, p 0.03) lags (S2 Fig, S2 Table). These correlations indicate lower relative salinities approximately four months after higher ONI values (El Niño periods) and higher relative salinities approximately four months after low ONI values (La Niña periods). Water temperature, dissolved oxygen and pH were not correlated with ONI, whether lagged or not (|r| ≤ 0.25).

### Reef development

**Fig 3:**
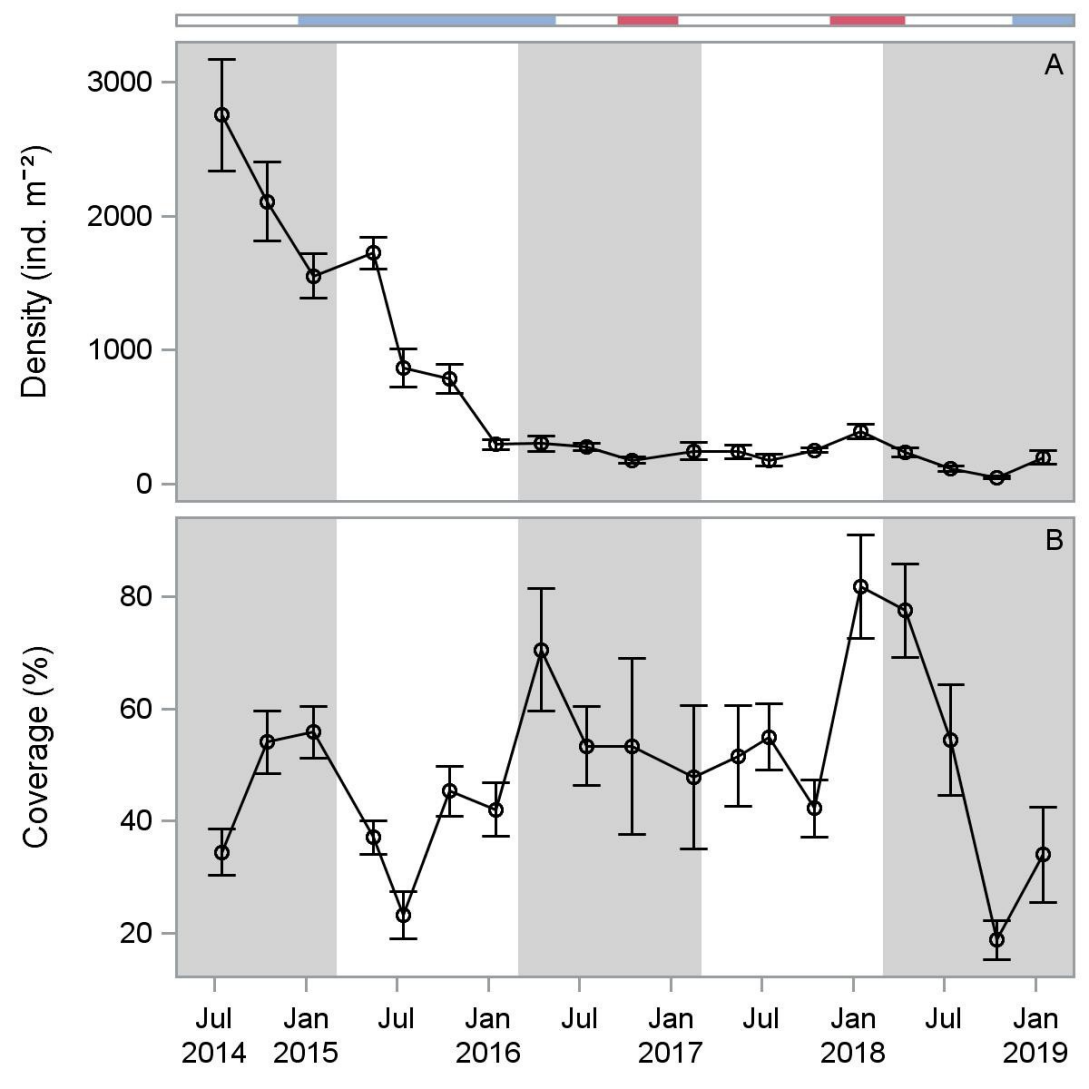
Mean oyster density (A) and percent areal coverage of reef substrates (B) on constructed sites from July 2014-January 2019. Colors on horizontal bar indicate ENSO state: light blue = El Niño, red = La Niña.

**Fig 4:**
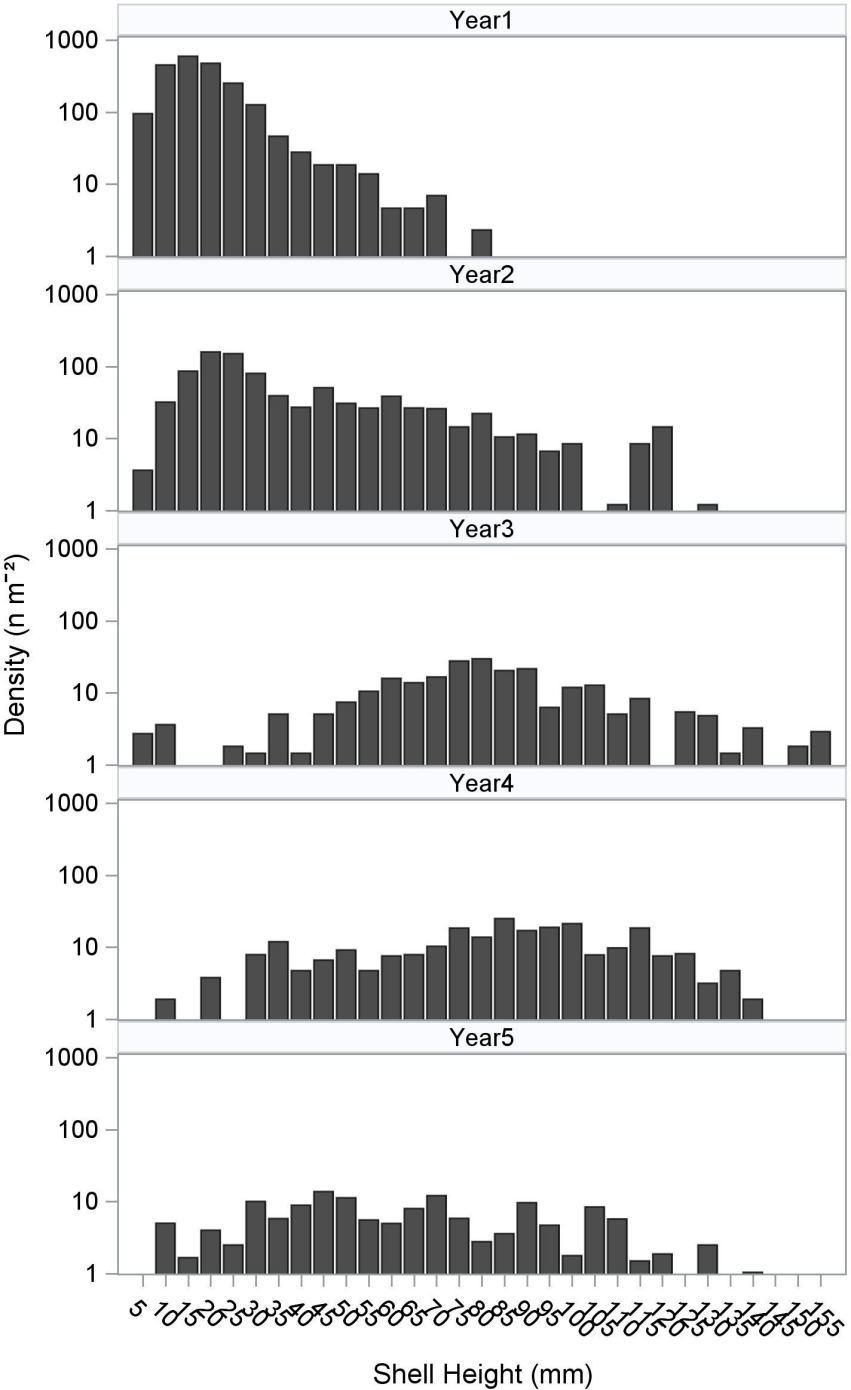
Size frequency distribution of oysters at constructed sites in years 1, 2, 3, 4, and 5 after reef construction.

Oyster density on the constructed reef decreased through time, with the highest mean density (2141 ind. m^-2^, all size classes) in the first year after reef construction, and the lowest mean density (151 ind. m^-2^, all size classes) in the fifth year (Fig 3A). Oyster density was positively correlated with ONI at 3-month (r = 0.54, p ≤ 0.03), 4-month (r = 0.59, p ≤ 0.02), 5-month (r = 0.55, p ≤ 0.03), and 6-month (r = 0.57, p ≤ 0.02) lags (S3 Fig, S3 Table). Mean areal coverage of constructed reef substrates by oysters ranged from 36.9 ± 2.7% in the second year to 57.2 ± 4.7% in the fourth year after reef construction (Table 1, Fig 3B). Areal coverage was highest in year 3 and 4, corresponding to high proportions of larger (submarket and market size) oysters on the reef. Areal coverage of constructed reef substrates was positively correlated with salinity (r = 0.62, p ≤ 0.01) but not ONI. Initial increases in coverage due to the arrival of new recruits were lost in year 2, which coincided with ENSO-driven reductions in salinity (Fig 3). Areal coverage declined again in year 5 with strong reductions in salinity. There was a shift from smaller to larger size classes of oysters in years 1–3, followed by a lack of recruitment in years 3–4, before transitioning back to a new set of recruits in year 5 (Fig 4, S4 Fig). Density of spat oysters (< 25 mm) was highest (1763 ± 247 ind. m^-2^) in the first year, and consistently low in the third through fifth years after reef construction (6–12 ind. m^-2^, Table 1). Submarket size (25–75 mm) oysters were most abundant in year 2 (423 ± 62 ind. m^-2^) and lower in years 3–5 (81–96 m^-2^). Market size (≥ 76 mm) oysters were present at the lowest density (2 ± 2 ind. m^-2^) in the first year after reef construction and the highest density in the fourth year (167 ± 18 ind. m^-2^).

**Table 1:** Annual mean (SE) oyster density, percent areal coverage by oysters, and P. marinus infection in years 1, 2, 3, 4, and 5 years after reef construction.

	Oyster density (ind. m^-2^)					*Perkinsus marinus* infection		
Year after construction	All size classes	Spat	Submarket	Market	Areal cover	Prevalence	Infection Intensity	Weighted Prevalence
1	2141 (206)	1763 (247)	376 (70)	2 (2)	48.1 (3.5)	4 (4)	0.2 (0.2)	0.1 (0.1)
2	883 (117)	365 (96)	423 (62)	88 (19)	36.9 (2.7)	0.6 (0.6)	0.0 (0.0)	0.0 (0.0)
3	250 (24)	6 (6)	96 (12)	147 (18)	56.6 (5.9)	0.0 (0.0)	0.0 (0.0)	0.0 (0.0)
4	262 (26)	6 (4)	81 (16)	167 (18)	57.2 (4.7)	31.4 (7.9)	0.4 (0.1)	0.3 (0.1)
5	151 (22)	12 (7)	87 (16)	47 (9)	46.2 (6.2)	30.0 (7.3)	0.2 (0.1)	0.1 (0.1)

Oyster spat <25 mm shell height, submarket >25–75 mm shell height, market ≥76 mm shell height. Areal coverage and prevalence are in %.

Prevalence of *P*. *marinus* infection in oysters on the constructed reef increased over time (Table 1, S5 Fig). Infection was largely absent from the constructed reef until the fourth and fifth years after reef construction, when an average of 31% and 30% of oysters were infected, respectively. Weighted prevalence of *P*. *marinus* infection remained at, or near zero for all years, ranging from 0.0 ± 0.0 in years 2 and 3, to 0.3 ± 0.1 in year 4.

### Habitat provision

**Fig 5:**
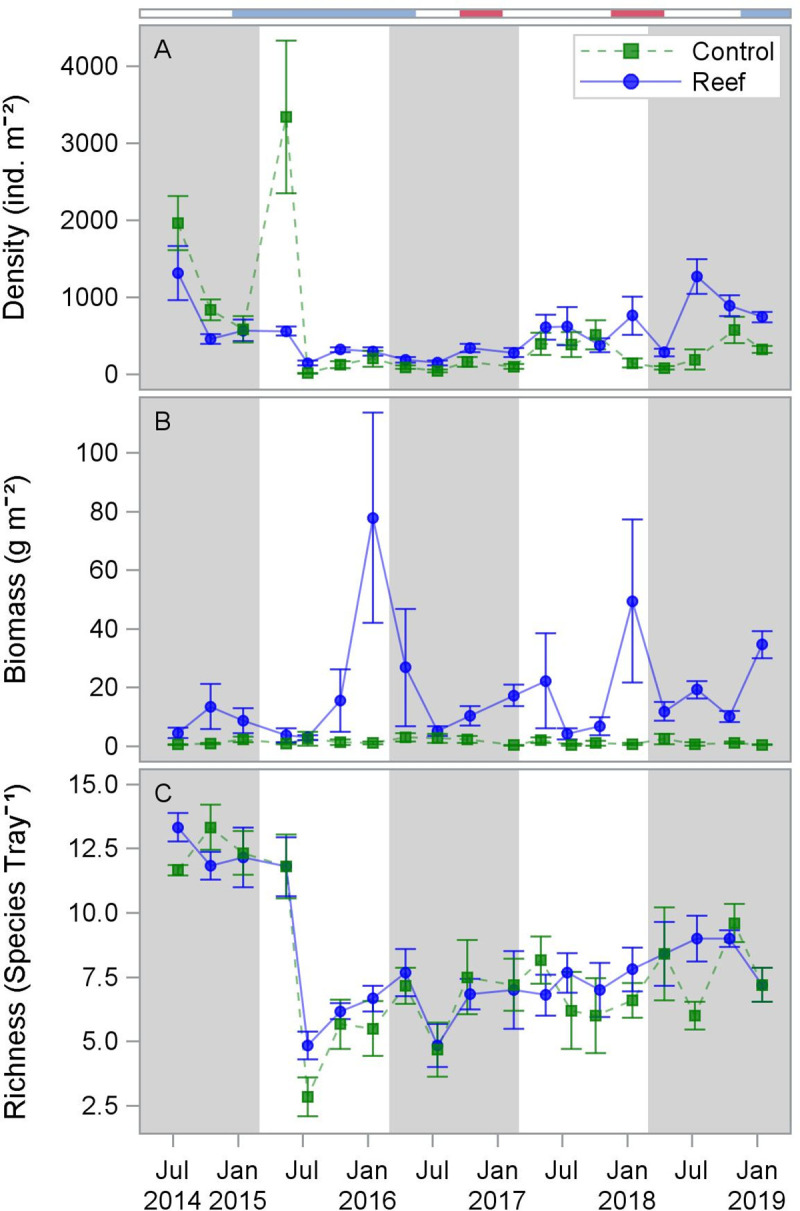
Mean (SE) resident motile fauna density (A), biomass (B), and richness (C) measured at constructed reef and control sites from July 2014-January 2019. Colors on horizontal bar indicate ENSO state: light blue = El Niño, red = La Niña.

Resident motile fauna density was generally lower on the reef than at the control sites for the first four sampling months and slightly higher thereafter (Fig 5A). Faunal density on the constructed reef was highest and most variable in the first (782 ± 151 ind. m^-2^) and fifth (798 ± 102 ind. m^-2^) years after reef construction, and lowest in the third year (240 ± 27 ind. m^-2^) after reef construction (Table 2). At control sites, faunal density was highest (1127 ± 195 ind. m^-2^) in the first year and lowest (102 ± 20 ind. m^-2^) in the third year after reef construction. An extraordinarily large recruitment of *Astyris* sp. dove snails into the control area caused the isolated peak in resident motile fauna density in May 2015 (2021 ind. m^-2^). The most abundant species at the constructed sites was the decapod *Petrolisthes* sp. (210 ± 26 ind. m^-2^, 40% of total), and at the control sites was the gastropod *Astyris* sp. (260 ± 69 ind. m^-2^, 50% of total; 150 ± 36 ind. m^-2^ not including peak in May 2015; S1 Table).

**Table 2:** Annual mean (SE) resident motile fauna density (ind. m^-2^), biomass (g m^-2^) and richness (species tray^-1^) at constructed reef and control sites in years 1, 2, 3, 4, and 5 after reef construction.

	Density		Biomass		Richness	
Year after construction	Reef	Control	Reef	Control	Reef	Control
1	782 (151)	1127 (195)	9.0 (3.0)	1.3 (0.4)	12 (1)	12 (1)
2	322 (37)	816 (347)	26.0 (11.3)	1.6 (0.6)	7 (1)	6 (1)
3	241 (27)	103 (20)	14.8 (5.3)	2.2 (0.6)	7 (1)	7 (1)
4	599 (110)	354 (72)	18.0 (7.2)	1.2 (0.4)	7 (1)	7 (1)
5	798 (103)	294 (66)	19.0 (2.7)	1.3 (0.5)	8 (1)	8 (1)

Biomass of resident motile fauna was higher and had greater variability at constructed reef sites than at control sites (Fig 5B). At constructed reef sites, biomass of resident motile fauna was highest (25.9 ± 11.3 g m^-2^) in the second year and lowest (9.0 ± 2.7 g m^-2^) in the first year after reef construction (Table 2). Biomass at control sites did not vary substantially, but was highest (2.2 ± 0.6 g m^-2^) in the third year and lowest (1.2 ± 0.4 g m^-2^) in the fourth year after reef construction. Presence of large adult *M*. *adina* stone crabs drove the peaks in biomass observed in winter samples at constructed reef sites (S8 Fig). Biomass at both the constructed reef (10.8 ± 2.9 g m^-2^, 61% of total) and control sites (0.25 ± 0.11 g m^-2^, 16% of total) was dominated by the decapod *M*. *adina* (S1 Table). There was no relationship between the dominant potential oyster predators [55] Xanthid crabs or *M*. *adina* with ONI or salinity.

Species richness was similar at the constructed reef and control sites throughout the study period (Fig 5C). Species richness was highest at both constructed reef and control sites (both averaging 12 species 0.28-m^-2^) in the first year after reef construction (Table 2). Species richness at both constructed reef and control sites then demonstrated a step-change decrease during the second year after reef construction, coincident with a large reduction in salinity, and remained low for the remainder of the study.

Resident motile fauna density was negatively correlated with ONI at 4-month (r = -0.53, p ≤ 0.02), 5-month (r = -0.56, p ≤ 0.01), and 6-month (r = -0.63, p ≤ 0.01) lags, and weakly but positively correlated with salinity (r = 0.40, p ≤ 0.10) (S6 Fig, S4 Table). Species richness was also negatively correlated with ONI at 5-month (r = -0.48, p ≤ 0.04), and 6-month (r = -0.46, p ≤ 0.05) lags. Species richness was positively correlated with salinity (r = 0.60, p ≤ 0.01). Resident motile fauna biomass was negatively correlated with temperature (r = -0.64, p ≤ 0.01) and positively correlated with dissolved oxygen (r = 0.63, p ≤ 0.01).

**Fig 6:**
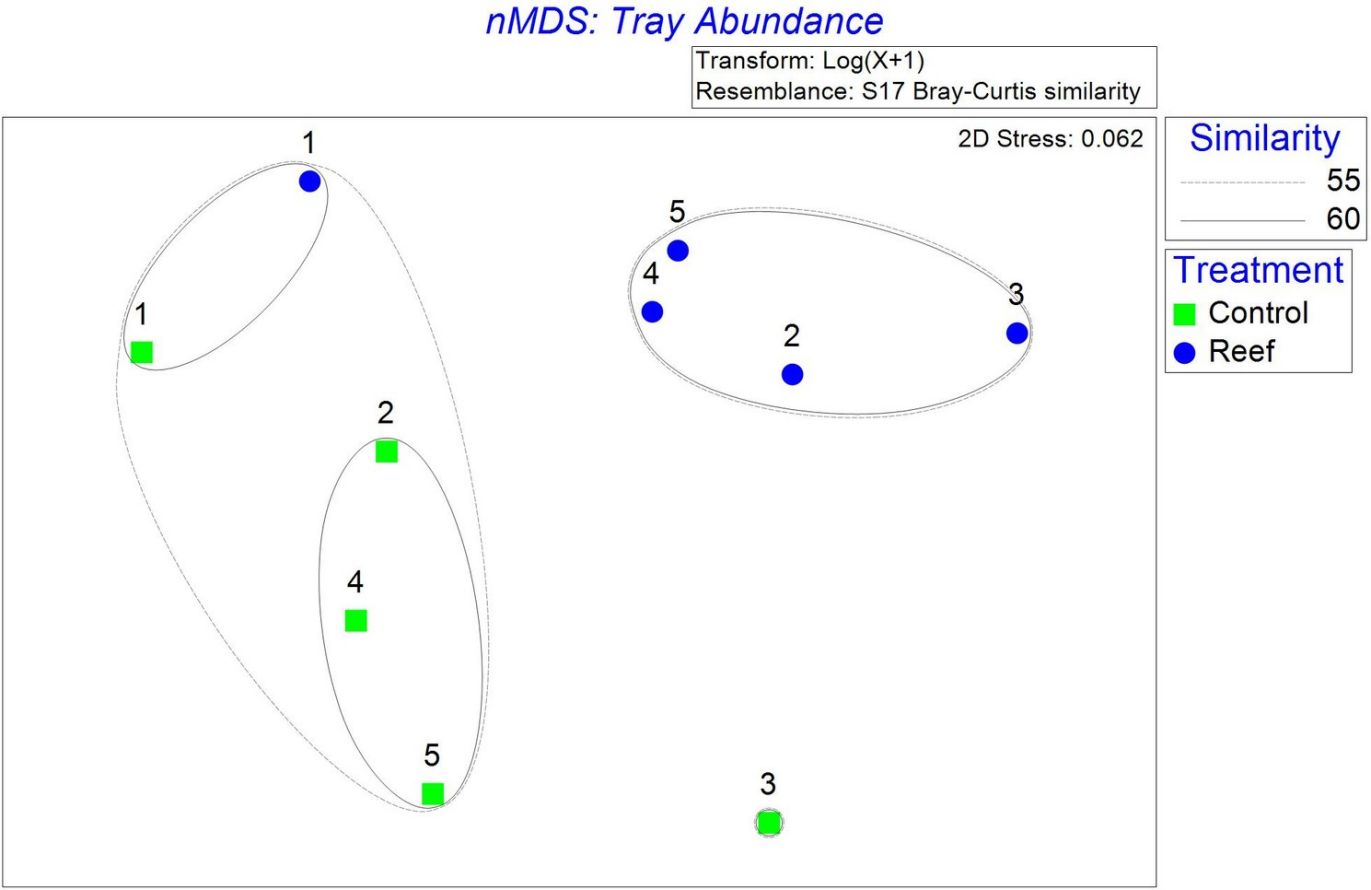
nMDS plot of resident motile fauna communities at constructed reef and control sites in years 1, 2, 3, 4, and 5 years after reef construction. Ellipses in the plot represent percent similarity of communities from cluster analysis.

Resident motile fauna community composition separated into three main groups with at least 55% similarity (p < 0.05; Fig 6). Community composition was similar at constructed reef and control sites during the first year after reef construction and became distinct by treatment in the second year and remained different through the end of the study (Fig 6). The multivariate community composition of the constructed reef fauna is most highly correlated with the combinations of 5-month lagged ONI and salinity (r = 0.399, p ≤ 0.023), 4-month lagged ONI and salinity (r = 0.377, p ≤ 0.033), and 6-month lagged ONI and salinity (r = 0.372, p ≤ 0.04). The single abiotic variable that is most highly correlated with community composition is salinity (r = 0.333, p ≤ 0.033), followed by 4-month lagged ONI (r = 0.278, p ≤ 0.083) and 5-month lagged ONI (r = 0.277, p ≤ 0.08).

## Discussion

Natural, self-sustainability of constructed oyster reefs depends on a continuous process of oyster recruitment, survival, and growth to ensure oyster population persistence in the face of mortality and sustain the ecological function of habitat provision for resident fauna. This process, in turn can be influenced by local-scale environmental variability and regional-scale climate variability operating over short- and long-timescales, which can only be understood after analyzing sustained, long-term measurements [11, 13, 14, 56, 57]. The five years of measurements following reef construction encompassed alternating El Niño and La Niña periods over approximately two-year cycles, both of which are known to influence precipitation and salinity in the study region [10, 58]. ENSO cycles were associated with changes in environmental and biotic variables at Half Moon Reef; ONI was negatively correlated with salinity and elicited changes over 2–5 month timeframes, similar to the 4–6 months reported by [13], who used cross-correlation analysis across all seven major estuaries along the Texas coast. Reductions in salinity and/or increases in ONI values in this study were linked to increases in oyster recruitment and density, decreases in areal coverage and resident motile fauna density and diversity, and shifts in faunal community composition. Results indicate that, when possible, sustained measurements of restoration projects should occur beyond the typical 1–2 years in order to assess the effects of ENSO-driven environmental variability more adequately. The time frame to capture ENSO-driven variability should be at least that of an ENSO cycle (approximately two to seven years).

### Reef development

Environmental and climate variability influenced oyster population dynamics throughout the five-year study. In the first two years following reef construction, oyster recruitment was rapid and new recruits (spat) composed 82% and 40% of all oysters on Half Moon Reef. Initial recruits likely benefited from clean substrates with relatively little fouling by organisms or sediments that can limit larval settlement of oyster larvae [59, 60] and possibly fewer predators. Despite gregarious recruitment in the first year and sustained recruitment in the second year, there was a conspicuous absence of new recruits (comprising only 3% and 2% of total in years 3 and 4) until the fifth year after reef construction (9% of total). Oyster density was positively correlated with ONI, and recruitment variability was likely influenced by ENSO-effects on local salinity (ONI is negatively correlated with local salinity), which in turn is known to influence oyster densities and recruitment dynamics in Gulf of Mexico estuaries [36, 37]. Higher and less temporally-variable salinities during years 3–4 coincided with increases in areal coverage of substrates by oysters, with >96% of oysters comprising larger size classes (submarket + market sizes) during the same period.

Interspecific and intraspecific interactions likely also contributed to oyster population dynamics on the constructed reef. High post-settlement mortality of oysters immediately following reef construction appeared to be density dependent as is characteristic of oyster populations when recruitment is high [61]. High densities of predatory decapod crustaceans (e.g., mud and stone crabs observed on the constructed reef (S7 and S8 Figs) indicate that predation may have also played a role in the high post-settlement mortality [55]. There was no influence of ONI or salinity on the dominant potential oyster predators Xanthid crabs or *M*. *adina*; however, because of their seasonality on the constructed reef, additional research across longer time scales would allow for an improved understanding of these relationships.

Infection of oysters on the constructed reef by *P*. *marinus* parasites was essentially zero until the fourth year after reef construction, which coincides with low ONI values and the occurrence of neutral and La Niña ENSO conditions from mid-year 3 to mid-year 5. La Niña conditions generally correspond to warmer, drier weather conditions in the Gulf of Mexico [10], which can create favorable conditions for *P*. *marinus*: higher water temperatures and salinities [40]. Our quarterly sampling frequency does not enable the determination of any interannual differences in water temperature at Half Moon Reef, nor do we observe any major temporal differences at a nearby monitoring station [47]. However, salinities are generally higher during the low ONI period (neutral and La Niña periods) from mid-year 3 to mid-year 5 than in year 2, when submarket and market-sized oysters become more dominant. Despite the onset of *P*. *marinus* infection coinciding with the onset of La Niña conditions, the delayed spread to the reef and low intensity at Half Moon Reef compared to other reefs with lower salinities [62] means that ENSO only partially explains the development of *P*. *marinus* infection at Half Moon Reef.

*Perkinsus marinus* infections are primarily spread by proximity to feces, pseudofeces, and decomposing tissues of infected oysters [63], and are generally slow to spread to new areas [64], although can be transmitted considerable distances from known sources [65]. In addition to environmental conditions, it is possible that the geographic isolation of Half Moon Reef from existing oyster reefs (~ 10 km) and lack of harvest or management activities that would introduce oysters from outside areas has delayed the spread of *P*. *marinus* to Half Moon Reef, and progression in the constructed reef oyster population. Additional research is warranted to assess the role of geographic isolation in slowing *P*. *marinus* disease transmission to constructed reefs as a strategy to reduce the potential for severe infection-related reductions in the oyster population [66]. It is likely that infections will continue to persist on Half Moon Reef because *P*. *marinus* is prevalent on reefs throughout Matagorda Bay and the Gulf of Mexico [36, 50, 62, 67], and infections tend to increase with oyster age [68]. However, ENSO-driven periodicity may serve to moderate infections through local reductions in salinity that can reduce *P*. *marinus* prevalence and severity [37, 40, 69].

### Habitat provision

The constructed reef provided immediate habitat benefits to resident motile fauna, which was associated with ENSO-driven effects on local salinity. In the first year after reef construction, resident motile faunal community composition and species richness were similar to those in the control area, indicating linkages between the constructed reef and surrounding unstructured habitat [70]. Coincident with reductions in salinity and oyster density in year 2, faunal community composition became distinct between the constructed reef and control sites, and overall fauna density decreased by about half, except for a large recruitment of the gastropod *Astyris* sp. in April 2015 to control sites. As the size frequency distribution shifted toward larger oysters, the constructed reef continued to support distinct faunal assemblages compared to the controls, however, motile fauna densities did not increase over time. The increase in oyster size equates to an increase in habitat complexity. Although more structurally complex habitats may be expected to sustain higher densities of organisms [71, 72], densities of resident motile fauna did not increase over time as the reef developed, indicating that reef presence, not complexity, was the most important factor affecting motile faunal communities in this study, as has been determined elsewhere [73, 74]. It is also possible that the extraordinary complexity introduced by reef construction (1.5 m height) provided much greater effects on faunal density than could be detected from any additional complexity produced by oyster growth (max oyster size = 168 mm) within the five-year study. Regardless, nMDS results indicate that a unique community of resident motile fauna was supported by the constructed reef by the second year after construction, thereby enhancing local biodiversity.

### Implications for restoration monitoring

Although typical funding cycles prioritize short-term ecological monitoring, longer term studies are critical for understanding responses to large-scale estuarine variability [30, 31], managing complex habitats [75], and informing restoration practices [76]. This five-year study captured more than one short ENSO cycle; however, ENSO cycles occur irregularly and, along with some other climate cycles (e.g. North Atlantic Oscillation, 3–6 year cycle; Pacific Decadal Oscillation, 15–25 year cycle), can occur over longer periods. Minimum periods for restoration monitoring should incorporate the periodicity of climate cycles that affect ecological dynamics (e.g., 2–7 years to capture ENSO-driven variability). Our results indicate that it is important to expand the typical monitoring timeframes to at least five years so that resource managers and restoration practitioners can better predict the long-term success of a restoration project. Our results also indicate the importance of seasonal sampling to capture temporal ecological cycles that would otherwise be missed. Future research is warranted to analyze restoration monitoring data collected over multiple climate cycles and from restoration projects implemented at different times within climate cycles to determine the best times to restore, monitor, and evaluate restoration success.

## Supporting information

S1 Table Mean (SE) resident motile fauna density (ind. m^-2^), biomass (g m^-2^), relative proportion (R%), and total catch, from constructed reef and control sites from July 2014-January 2019. (PDF)

S2 Table Spearman correlation results for ONI (with lags of 1-month (lagONI), 2-month (lag2ONI), 3-month (lag3ONI), 4-month (lag4ONI), 5-month (lag5ONI), and 6-month (lag6ONI)) and water quality variables (Temp = temperature, DO = dissolved oxygen). See S2 Fig for matrix plot of results.
(PDF)

S3 Table Spearman correlation results for mean oyster density (nm2_Mean) and percent areal coverage (percov_Mean) with water quality variables (Temp = temperature, DO = dissolved oxygen), and ONI (with lags of 1-month (lagONI), 2-month (lag2ONI), 3-month (lag3ONI), 4-month (lag4ONI), 5-month (lag5ONI), and 6-month (lag6ONI)). See S3 Fig for matrix plot of results.
(PDF)

S4 Table Spearman correlation results for motile fauna density, biomass, N1 diversity and species richness with water quality variables (Temp = temperature, DO = dissolved oxygen), and ONI (with lags of 1-month (lagONI), 2-month (lag2ONI), 3-month (lag3ONI), 4-month (lag4ONI), 5-month (lag5ONI), and 6-month (lag6ONI)). See S6 Fig for matrix plot of results.
(PDF)

S1 Fig Mean temperature (°C; A), dissolved oxygen (mg l-1; B) and pH (C) at the surface (< 1 m depth) and bottom (> 1 m depth) at Half Moon Reef, measured quarterly from January 2014-January 2019.
(TIF)

S2 Fig Spearman correlation figures for ONI (with lags of 1-month (lagONI), 2-month (lag2ONI), 3-month (lag3ONI), 4-month (lag4ONI), 5-month (lag5ONI), and 6-month (lag6ONI)) and water quality variables (Sal = salinity, Temp = temperature (°C), DO_mgL = dissolved oxygen (mg l^-1^)). See S2 Table for r and p-values. Plots depicting negative relationships are outlined with red (r ≤ -0.50), orange (r ≤ -0.45) and yellow (r ≤ -0.40).
(TIF)

S3 Fig Spearman correlation figures for mean oyster density and percent areal coverage with water quality variables (Temp = temperature, DO = dissolved oxygen), and ONI (with lags of 1-month (lagONI), 2-month (lag2ONI), 3-month (lag3ONI), 4-month (lag4ONI), 5-month (lag5ONI), and 6-month (lag6ONI)). See S3 Table for r and p-values. Plots depicting negative relationships are outlined with red (r ≤ -0.50), orange (r ≤ -0.45) and yellow (r ≤ -0.40). Plots depicting positive relationships are outlined with dark green (r ≥ 0.60), medium green (r ≥ 0.50) and light green (r ≥ 0.40).
(TIF)

S4 Fig Cumulative oyster density (n m^-2^) and size-specific oyster density (n m^-2^) for spat (<25 mm), submarket (>25–75 mm) and market (≥ 76 mm) sized oysters at Half Moon Reef, measured quarterly from July 2014-January 2019. (TIF)

S5 Fig Proportion of oysters infected with P. marinus (prevalence) and severity of infection (weighted prevalence) on Half Moon Reef. Blue shading indicates one standard deviation on either side of the mean. The maximum weighted prevalence possible is 5.
(TIF)

S6 Fig Spearman correlation figures for motile fauna density, biomass, N1 diversity and species richness with water quality variables (Temp = temperature, DO = dissolved oxygen), and ONI (with lags of 1-month (lagONI), 2-month (lag2ONI), 3-month (lag3ONI), 4-month (lag4ONI), 5-month (lag5ONI), and 6-month (lag6ONI)). See S4 Table for r and p-values. Plots depicting negative relationships are outlined with dark red (r ≤ -0.60), red (r ≤ -0.50), orange (r ≤ -0.45) and yellow (r ≤ -0.40). Plots depicting positive relationships are outlined with dark green (r ≥ 0.60), medium green (r ≥ 0.50) and light green (r ≥ 0.40).
(TIF)

S7 Fig Mean (SE) Xanthidae density (A) and biomass (B), and salinity and ONI (C) measured quarterly from January 2014-January 2019. ONI data are from [18].
(TIF)

S8 Fig Mean (SE) *Menippe adina* density (A) and biomass (B), and salinity and ONI (C) measured quarterly from January 2014-January 2019. ONI data are from [18].
(TIF)
