# Supplementary material for: Medium-term monitoring reveals effects of El Niño Southern Oscillation climate variability on local salinity and faunal dynamics on a restored oyster reef
Source: PLoS One. 2021 Aug 16;16(8):e0255931. doi: 10.1371/journal.pone.0255931 (PMC8366962; doi:10.1371/journal.pone.0255931)
Supplement: S1 Table — (PDF) [file pone.0255931.s001.pdf]

Tray species list Densities listed as mean (standard error). Standard error is calculated from all dates and stations

		Resident motile fauna density (ind m^{-2})				Resident motile fauna biomass (g m^{-2})				Total Catch (n)	
Higher Taxa	Taxa	Reef	R%	Control	R%	Reef	R%	Control	R%	Reef	Control
Decapoda	Petrolisthes sp.	210.86 (26.34)	39.7	37.45 (6.55)	7.2	2.81 (0.33)	15.9	0.21 (0.03)	0.0	6390	1093
Gastropoda	Panopeus herbstii	89.33 (10.5)	16.8	10.49 (1.74)	2.0	1.76 (0.27)	10.0	0.08 (0.02)	0.0	2707	306
Gastropoda	Parvanachis ostreicola	79.79 (19.26)	15.0	92.62 (17.48)	17.7	0.04 (0.01)	0.2	0.03 (0.01)	0.0	2418	2703
Decapoda	Xanthidae	56.59 (7.73)	10.7	18.33 (3.38)	3.5	0.37 (0.1)	2.1	0.10 (0.04)	0.0	1715	535
Gastropoda	Costoanachis sp.	25.77 (5.42)	4.9	32.14 (10.97)	6.1	0.09 (0.02)	0.5	0.04 (0.01)	0.0	781	938
Gastropoda	Astyris sp.	20.49 (4.51)	3.9	259.75 (69.36)	49.7	0.01 (0.00)	0.0	0.09 (0.03)	0.0	621	7580
Decapoda	Menippe adina	12.41 (1.86)	2.3	2.47 (0.65)	0.5	10.8 (2.89)	61.0	0.25 (0.11)	0.0	376	72
Decapoda	Alpheus heterochaelis	6.8 (0.98)	1.3	1.03 (0.33)	0.2	0.32 (0.05)	1.8	0.01 (0.00)	0.0	206	30
Decapoda	Synalpheus fritzmuelleri	4.32 (1.22)	0.8	0.86 (0.39)	0.2	0.01 (0.00)	0.1	0.00 (0.00)	0.0	131	25
Decapoda	Eurypanopeus sp.	4.26 (1.67)	0.8	0.27 (0.13)	0.1	0.22 (0.09)	1.3	0.02 (0.01)	0.0	129	8
Decapoda	Paguroidea	2.81 (0.54)	0.5	22.82 (4.63)	4.4	0.01 (0.00)	0.0	0.07 (0.04)	0.0	85	666
Decapoda	Palaemonetes sp.	2.38 (0.66)	0.4	2.84 (1.16)	0.5	0.05 (0.01)	0.3	0.03 (0.01)	0.0	72	83
Platyhelminthes	Platyhelminthes	1.85 (0.35)	0.3	2.09 (0.95)	0.4	0.01 (0.00)	0.0	0.00 (0.00)	0.0	56	61
Gastropoda	Triphora nigrocincta	1.72 (0.67)	0.3	1.37 (0.51)	0.3	0.00 (0.00)	0.0	0.00 (0.00)	0.0	52	40
Gastropoda	Stramonita haemastoma	1.32 (0.5)	0.2	0.58 (0.22)	0.1	0.62 (0.21)	3.5	0.22 (0.09)	0.0	40	17
Gastropoda	Aeolidiidae	1.29 (0.5)	0.2	10.97 (3.91)	2.1	0.00 (0.00)	0.0	0.00 (0.00)	0.0	39	320
Teleostei	Gobiosoma bosc	1.29 (0.33)	0.2	1.1 (0.32)	0.2	0.03 (0.02)	0.2	0.01 (0.00)	0.0	39	32
Decapoda	Diogenidae	1.19 (0.45)	0.2	2.54 (0.7)	0.5	0.18 (0.06)	1.0	0.23 (0.09)	0.0	36	74
Gastropoda	Boonea impressa	1.09 (0.32)	0.2	0.89 (0.54)	0.2	0.00 (0.00)	0.0	0.00 (0.00)	0.0	33	26
Decapoda	Pelia mutica	0.76 (0.23)	0.1	1.89 (0.52)	0.4	0.01 (0.00)	0.0	0.02 (0.01)	0.0	23	55
Gastropoda	Nassarius acutus	0.56 (0.19)	0.1	9.7 (4.16)	1.9	0.00 (0.00)	0.0	0.01 (0.01)	0.0	17	283
Decapoda	Dyspanopeus texanus	0.5 (0.33)	0.1	0.14 (0.14)	0.0	0.00 (0.00)	0.0	0.00 (0.00)	0.0	15	4
Gastropoda	Turbonilla sp.	0.5 (0.19)	0.1	1.44 (0.48)	0.3	0.00 (0.00)	0.0	0.00 (0.00)	0.0	15	42
Decapoda	Portunidae	0.46 (0.18)	0.1	1.78 (0.56)	0.3	0.00 (0.00)	0.0	0.01 (0.00)	0.0	14	52
Decapoda	Clibanarius vittatus	0.36 (0.13)	0.1	0.17 (0.12)	0.0	0.13 (0.05)	0.7	0.06 (0.04)	0.0	11	5
Teleostei	Opsanus beta	0.36 (0.1)	0.1	0 (0)	0.0	0.03 (0.01)	0.1	0 (0)	0.0	11	0
Peracarida	Mysidacea	0.3 (0.17)	0.1	0.69 (0.36)	0.1	0.00 (0.00)	0.0	0.00 (0.00)	0.0	9	20
Gastropoda	Epitonium sp.	0.23 (0.12)	0.0	0.86 (0.24)	0.2	0.00 (0.00)	0.0	0.00 (0.00)	0.0	7	25
Teleostei	Gobiidae larvae	0.23 (0.13)	0.0	0.79 (0.44)	0.2	0.00 (0.00)	0.0	0.00 (0.00)	0.0	7	23
Gastropoda	Pyrgocythara hemphilli	0.2 (0.14)	0.0	0 (0)	0.0	0.00 (0.00)	0.0	0 (0)	0.0	6	32
Teleostei	Gobiesox strumosus	0.2 (0.11)	0.0	0.03 (0.03)	0.0	0.05 (0.04)	0.3	0.00 (0.00)	0.0	6	0
Gastropoda	Acteocina canaliculata	0.2 (0.17)	0.0	1.1 (0.41)	0.2	0.00 (0.00)	0.0	0.00 (0.00)	0.0	6	1

Tray species list Densities listed as mean (standard error). Standard error is calculated from all dates and stations

		Resident motile fauna density (ind m^{-2})				Resident motile fauna biomass (g m^{-2})				Total Catch (n)	
Higher Taxa	Taxa	Reef	R%	Control	R%	Reef	R%	Control	R%	Reef	Control
Decapoda	Acetes americanus	0.13 (0.13)	0.0	0.1 (0.08)	0.0	0.00 (0.00)	0.0	0.00 (0.00)	0.0	4	3
Bivalvia	Ischadium recurvum	0.1 (0.06)	0.0	0 (0)	0.0	0.00 (0.00)	0.0	0 (0)	0.0	3	0
Decapoda	Heterocrypta granulata	0.1 (0.07)	0.0	0.24 (0.1)	0.0	0.00 (0.00)	0.0	0.00 (0.00)	0.0	3	7
Teleostei	Hypsoblennius hentz	0.1 (0.06)	0.0	0 (0)	0.0	0.14 (0.09)	0.8	0 (0)	0.0	3	0
Decapoda	Pinnixa sp.	0.07 (0.05)	0.0	0 (0)	0.0	0.00 (0.00)	0.0	0 (0)	0.0	2	0
Gastropoda	Pyrgocythara plicosa	0.07 (0.05)	0.0	0 (0)	0.0	0.00 (0.00)	0.0	0 (0)	0.0	2	0
Gastropoda	Siella adamsi	0.07 (0.05)	0.0	0.27 (0.11)	0.1	0.00 (0.00)	0.0	0.00 (0.00)	0.0	2	8
Decapoda	Post-larval penaid	0.03 (0.03)	0.0	0 (0)	0.0	0.00 (0.00)	0.0	0 (0)	0.0	1	0
Gastropoda	Cancellaria sp.	0.03 (0.03)	0.0	0 (0)	0.0	0.00 (0.00)	0.0	0 (0)	0.0	1	0
Gastropoda	Cymatium sp.	0.03 (0.03)	0.0	0 (0)	0.0	0.00 (0.00)	0.0	0 (0)	0.0	1	0
Gastropoda	Eulimastoma sp.	0.03 (0.03)	0.0	0.41 (0.19)	0.1	0.00 (0.00)	0.0	0.00 (0.00)	0.0	1	12
Gastropoda	Paradentalium americanum	0.03 (0.03)	0.0	0 (0)	0.0	0.00 (0.00)	0.0	0 (0)	0.0	1	0
Teleostei	Gobiidae	0.03 (0.03)	0.0	0.03 (0.03)	0.0	0.00 (0.00)	0.0	0.00 (0.00)	0.0	1	1
Bivalvia	Ensis minor	0 (0)	0	0.1 (0.08)	0.0	0 (0)	0.0	0.00 (0.00)	0.0	0	3
Decapoda	Callinectes similis	0 (0)	0	0.31 (0.12)	0.1	0 (0)	0.0	0.01 (0.01)	0.0	0	9
Decapoda	Callinectes sp.	0 (0)	0	0.07 (0.05)	0.0	0 (0)	0.0	0.00 (0.00)	0.0	0	2
Decapoda	Latreutes parvulus	0 (0)	0	0.03 (0.03)	0.0	0 (0)	0.0	0.00 (0.00)	0.0	0	1
Decapoda	Leptochela serratorbita	0 (0)	0	0.03 (0.03)	0.0	0 (0)	0.0	0.00 (0.00)	0.0	0	1
Decapoda	Penaeidae	0 (0)	0	0.03 (0.03)	0.0	0 (0)	0.0	0.00 (0.00)	0.0	0	1
Decapoda	Tozeuma carolinense	0 (0)	0	0.03 (0.03)	0.0	0 (0)	0.0	0.00 (0.00)	0.0	0	1
Echinodermata	Ophiurida	0 (0)	0	0.62 (0.25)	0.1	0 (0)	0.0	0.02 (0.01)	0.0	0	18
Gastropoda	Amaea mitchelli	0 (0)	0	0.34 (0.23)	0.1	0 (0)	0.0	0.00 (0.00)	0.0	0	10
Gastropoda	Cantharus cancellarius	0 (0)	0	0.31 (0.22)	0.1	0 (0)	0.0	0.02 (0.02)	0.0	0	9
Gastropoda	Cerithiopsis sp.	0 (0)	0	0.03 (0.03)	0.0	0 (0)	0.0	0.00 (0.00)	0.0	0	1
Gastropoda	Fargoa sp.	0 (0)	0	0.14 (0.08)	0.0	0 (0)	0.0	0.00 (0.00)	0.0	0	4
Gastropoda	Neverita duplicata	0 (0)	0	0.10 (0.10)	0.0	0 (0)	0.0	0.00 (0.00)	0.0	0	3
Gastropoda	Pyramidellidae	0 (0)	0	0.10 (0.08)	0.0	0 (0)	0.0	0.00 (0.00)	0.0	0	3
Gastropoda	Turridae	0 (0)	0	0.10 (0.06)	0.0	0 (0)	0.0	0.00 (0.00)	0.0	0	3
Gastropoda	Zebina sp.	0 (0)	0	0.07 (0.07)	0.0	0 (0)	0.0	0.00 (0.00)	0.0	0	2
Teleostei	Ctenogobius boleosoma	0 (0)	0	0.10 (0.08)	0.0	0 (0)	0.0	0.00 (0.00)	0.0	0	3
Teleostei	Cynoglossidae	0 (0)	0	0.07 (0.05)	0.0	0 (0)	0.0	0.00 (0.00)	0.0	0	2
Teleostei	Sciaenidae larvae	0 (0)	0	0.14 (0.14)	0.0	0 (0)	0.0	0.00 (0.00)	0.0	0	4

Tray species list   Densities listed as mean (standard error). Standard error is calculated from all dates and stations

		Resident motile fauna density (ind m^{-2})				Resident motile fauna biomass (g m^{-2})				Total Catch (n)	
Higher Taxa	Taxa	Reef	R%	Control	R%	Reef	R%	Control	R%	Reef	Control
	Sum	531.20	100.0	522.99		17.70	100.0	1.56	100.0	16098	15262
