# Supplementary material for: Medium-term monitoring reveals effects of El Niño Southern Oscillation climate variability on local salinity and faunal dynamics on a restored oyster reef
Source: PLoS One. 2021 Aug 16;16(8):e0255931. doi: 10.1371/journal.pone.0255931 (PMC8366962; doi:10.1371/journal.pone.0255931)
Supplement: S2 Table — See S2 Fig for matrix plot of results. (PDF) [file pone.0255931.s002.pdf]

Spearman Correlation Coefficients (r), N = 22<br>Prob > |r| under H0: r = 0 (p-value)								
**Variable**		**ONI**	**lagONI**	**lag2ONI**	**lag3ONI**	**lag4ONI**	**lag5ONI**	**lag6ONI**
**Salinity**	**r**	-0.18490	-0.31731	-0.43395	-0.48005	-0.50962	-0.47436	-0.40817
	p	0.4101	0.1502	0.0436	0.0238	0.0154	0.0257	0.0593
**Temp**	**r**	0.06503	0.16855	0.20368	0.18228	0.15102	0.09068	-0.01134
(°C)	p	0.7737	0.4534	0.3633	0.4168	0.5023	0.6882	0.9601
**DO**	**r**	-0.02771	-0.14823	-0.23316	-0.25368	-0.23197	-0.17064	-0.04678
(mg l^{-1})	p	0.9026	0.5103	0.2964	0.2546	0.2989	0.4477	0.8362
**pH**	**r**	-0.24484	-0.19457	-0.18274	-0.18002	-0.09615	0.04194	0.05952
	p	0.2721	0.3856	0.4156	0.4228	0.6704	0.8530	0.7925
