# Supplementary material for: Medium-term monitoring reveals effects of El Niño Southern Oscillation climate variability on local salinity and faunal dynamics on a restored oyster reef
Source: PLoS One. 2021 Aug 16;16(8):e0255931. doi: 10.1371/journal.pone.0255931 (PMC8366962; doi:10.1371/journal.pone.0255931)
Supplement: S3 Table — See S3 Fig for matrix plot of results. (PDF) [file pone.0255931.s003.pdf]

Spearman Correlation Coefficients (r), N = 16<br>Prob > |r| under H0: r = 0 (p-value)			
		**Density (n m^{-2})**	**Coverage (%)**
**Salinity**	**r**	-0.24412	0.62059
	p	0.3622	0.0103
**Temp**	**r**	-0.08529	0.03235
(°C)	p	0.7535	0.9053
**DO**	**r**	0.02941	0.08529
(mg l^{-1})	p	0.9139	0.7535
**pH**	**r**	0.15000	0.32941
	p	0.5792	0.2128
**ONI**	**r**	0.36819	-0.49632
	p	0.1606	0.0505
**lagONI**	**r**	0.42415	-0.45950
	p	0.1016	0.0734
**lag2ONI**	**r**	0.47241	-0.35320
	p	0.0646	0.1796
**lag3ONI**	**r**	0.53943	-0.34341
	p	0.0310	0.1928
**lag4ONI**	**r**	0.58997	-0.23304
	p	0.0161	0.3851
**lag5ONI**	**r**	0.54787	-0.09720
	p	0.0280	0.7203
**lag6ONI**	**r**	0.57480	-0.01326
	p	0.0199	0.9611
