# Supplementary material for: Medium-term monitoring reveals effects of El Niño Southern Oscillation climate variability on local salinity and faunal dynamics on a restored oyster reef
Source: PLoS One. 2021 Aug 16;16(8):e0255931. doi: 10.1371/journal.pone.0255931 (PMC8366962; doi:10.1371/journal.pone.0255931)
Supplement: S4 Table — See S6 Fig for matrix plot of results. (PDF) [file pone.0255931.s004.pdf]

Spearman Correlation Coefficients (r), N = 19 Prob > |r| under H0: r = 0 (p-value)					
		**Density (n m^{-2})**	**Biomass (g m^{-2})**	**Hill's N1 Diversity**	**Species Richness**
**Salinity**	**r**	0.39649	0.26491	0.59123	0.59666
	p	0.0928	0.2731	0.0077	0.0070
**Temp**	**r**	-0.00526	-0.63860	-0.18070	-0.18278
(°C)	p	0.9829	0.0033	0.4591	0.4539
**DO**	**r**	0.00439	0.62571	0.10443	0.13758
(mg l^{-1})	p	0.9858	0.0042	0.6705	0.5743
**pH**	**r**	0.14737	-0.13158	0.12105	-0.11599
	p	0.5471	0.5913	0.6215	0.6363
**ONI**	**r**	-0.15108	0.03250	0.01142	-0.15222
	p	0.5370	0.8949	0.9630	0.5339
**lagONI**	**r**	-0.26350	-0.04831	-0.03601	-0.25825
	p	0.2757	0.8443	0.8836	0.2857
**lag2ONI**	**r**	-0.37802	-0.09055	-0.08176	-0.36416
	p	0.1105	0.7124	0.7393	0.1253
**lag3ONI**	**r**	-0.44728	-0.09051	-0.08260	-0.37764
	p	0.0548	0.7125	0.7367	0.1109
**lag4ONI**	**r**	-0.53603	-0.07733	-0.08260	-0.44190
	p	0.0180	0.7530	0.7367	0.0582
**lag5ONI**	**r**	-0.56138	-0.05103	-0.07655	-0.47951
	p	0.0124	0.8356	0.7554	0.0378
**lag6ONI**	**r**	-0.63005	-0.00088	-0.06503	-0.46127
	p	0.0038	0.9972	0.7914	0.0468
