# Supplementary figures and images for: Medium-term monitoring reveals effects of El Niño Southern Oscillation climate variability on local salinity and faunal dynamics on a restored oyster reef
Source: PLoS One. 2021 Aug 16;16(8):e0255931. doi: 10.1371/journal.pone.0255931 (PMC8366962; doi:10.1371/journal.pone.0255931)

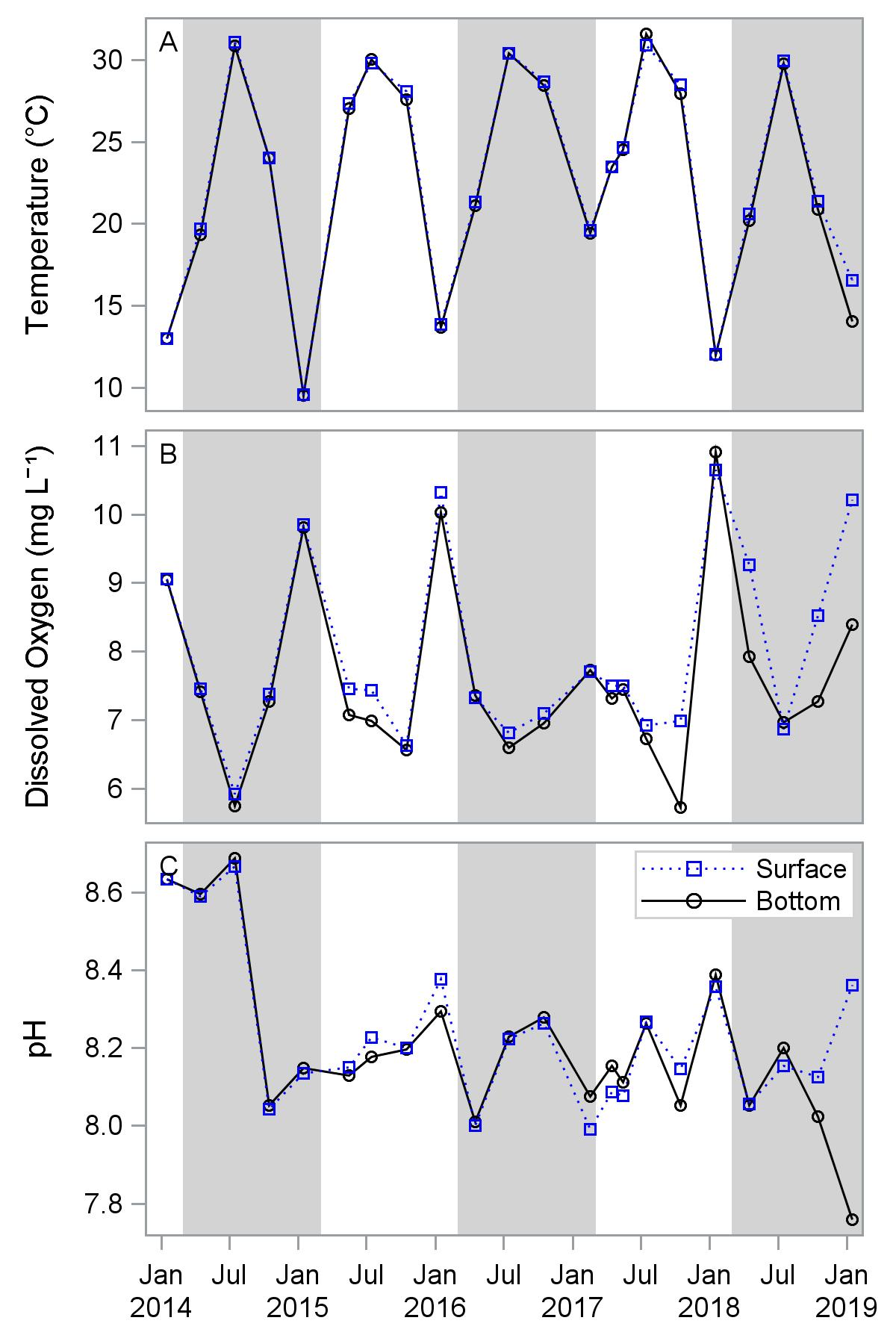

Supplement: S1 Fig — Mean temperature (°C; A), dissolved oxygen (mg l-1; B) and pH (C) at the surface (< 1 m depth) and bottom (> 1 m depth) at Half Moon Reef, measured quarterly from January 2014-January 2019. (TIF) [file pone.0255931.s005.tif]

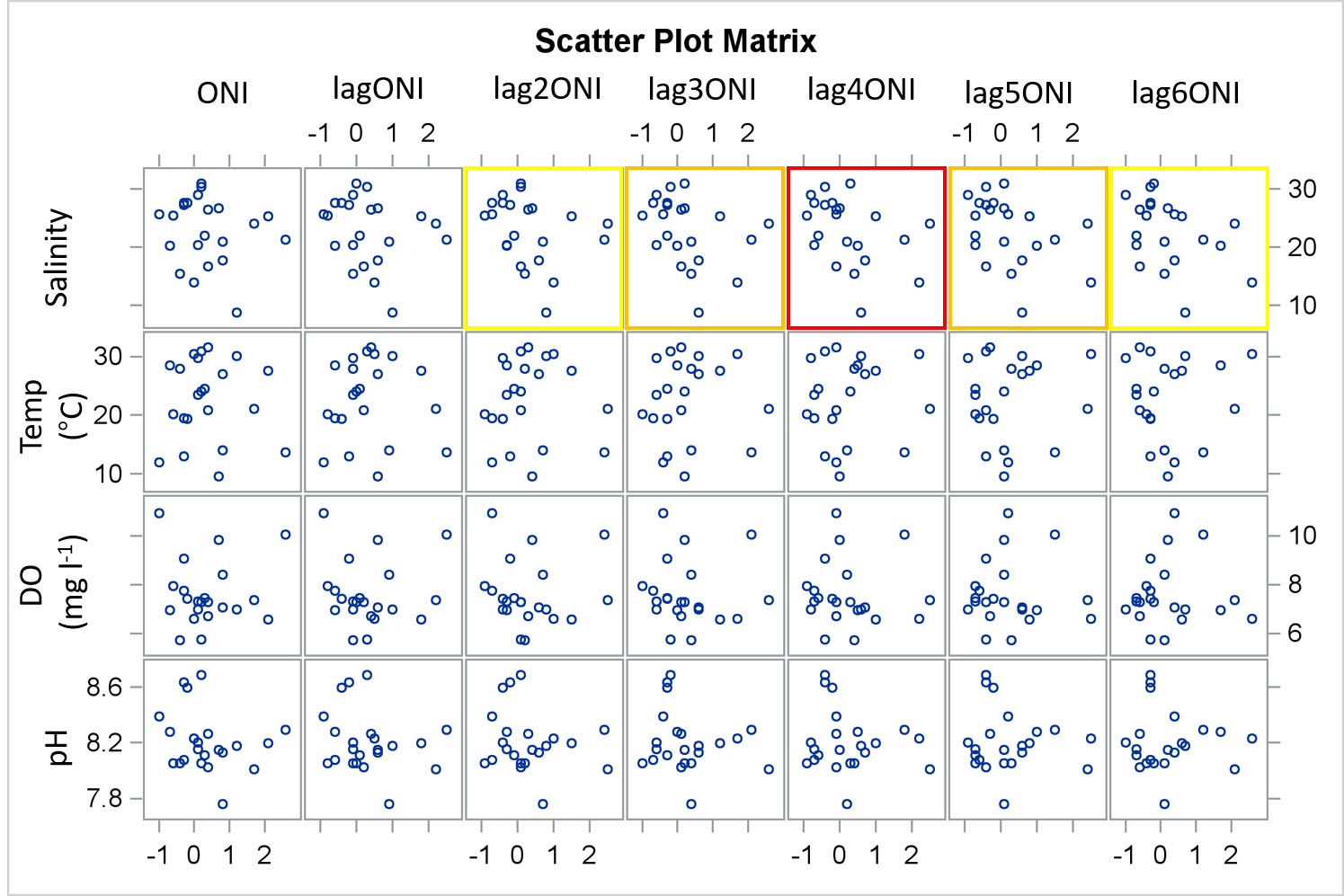

Supplement: S2 Fig — See S2 Table for r and p-values. Plots depicting negative relationships are outlined with red (r ≤ -0.50), orange (r ≤ -0.45) and yellow (r ≤ -0.40). (TIF) [file pone.0255931.s006.tif]

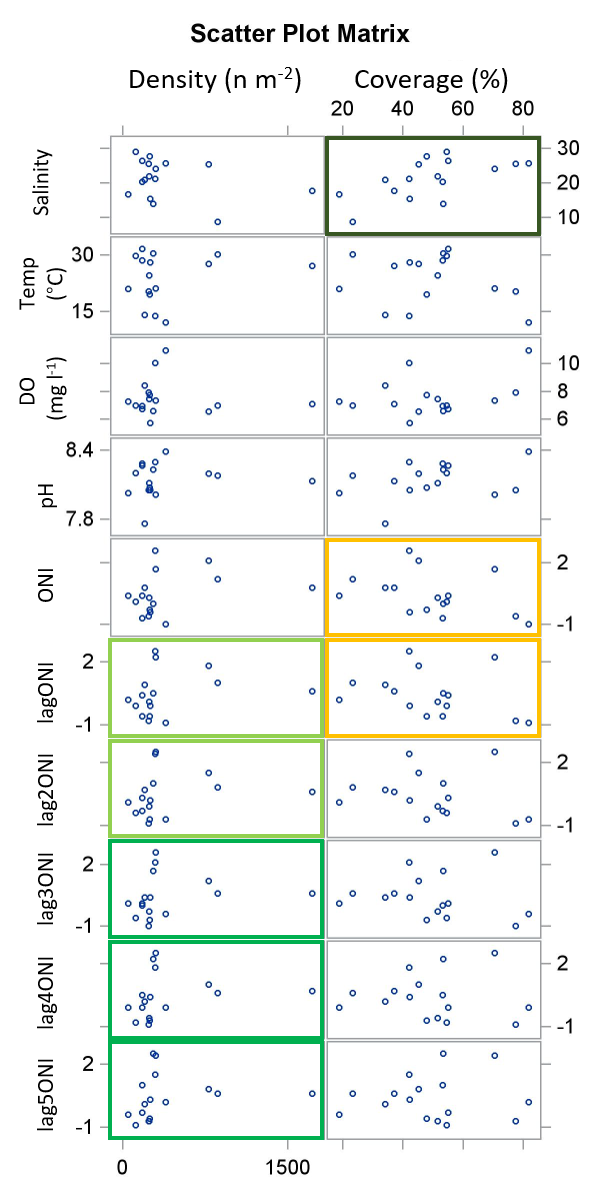

Supplement: S3 Fig — See S3 Table for r and p-values. Plots depicting negative relationships are outlined with red (r ≤ -0.50), orange (r ≤ -0.45) and yellow (r ≤ -0.40). Plots depicting positive relationships are outlined with dark green (r ≥ 0.60), medium green (r ≥ 0.50) and light green (r ≥ 0.40). (TIF) [file pone.0255931.s007.tif]

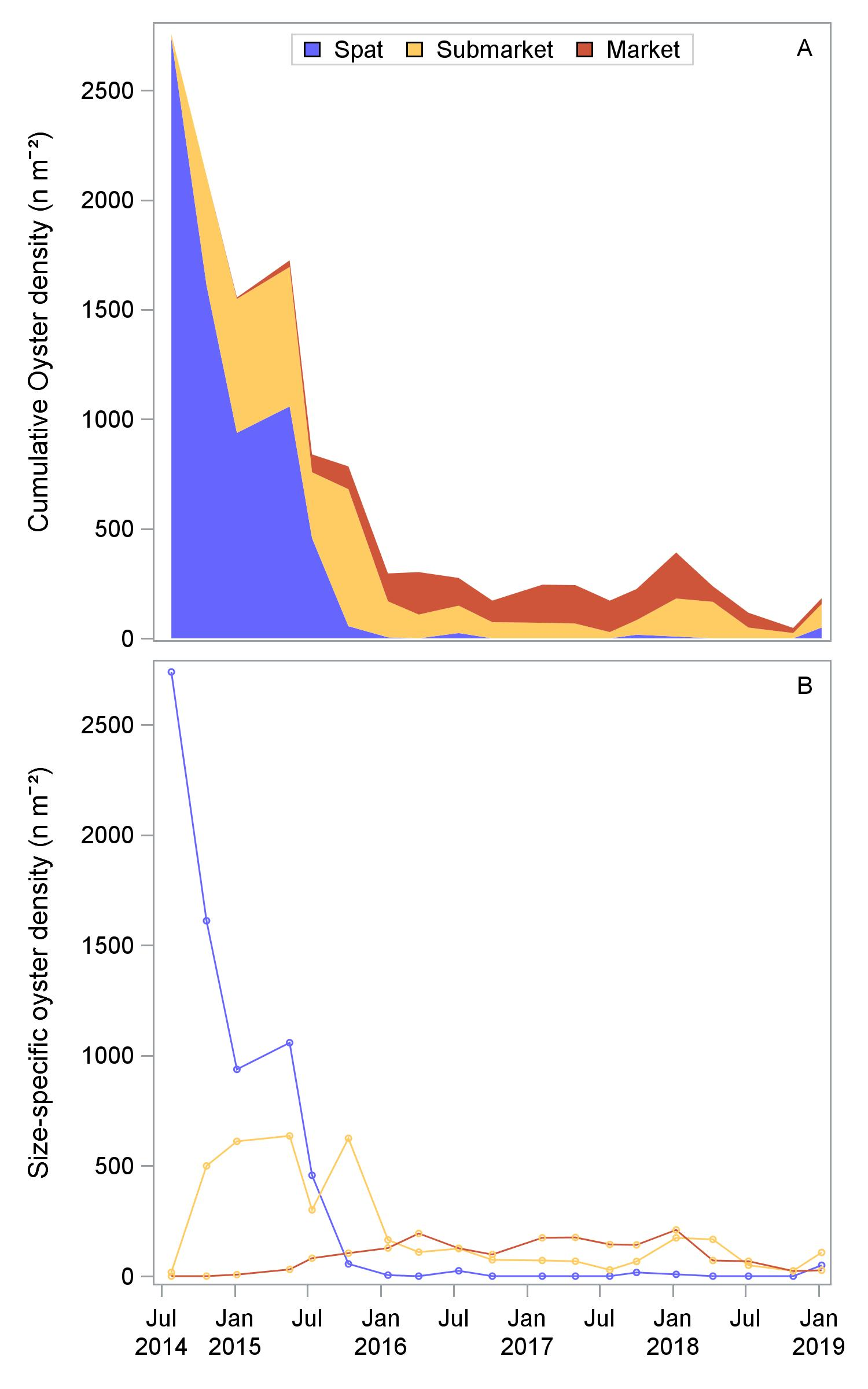

Supplement: S4 Fig — (TIF) [file pone.0255931.s008.tif]

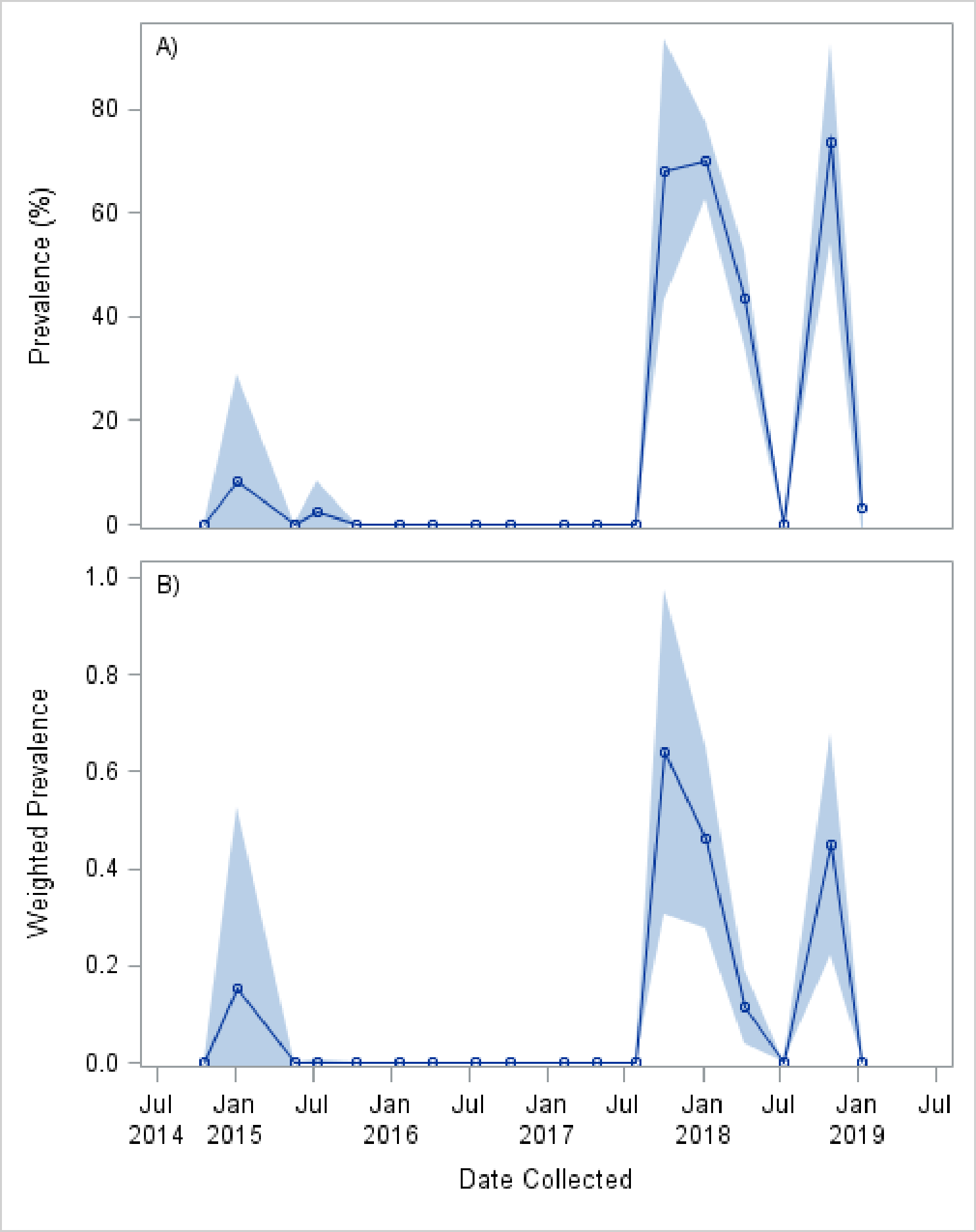

Supplement: S5 Fig — Blue shading indicates one standard deviation on either side of the mean. The maximum weighted prevalence possible is 5. (TIF) [file pone.0255931.s009.tif]

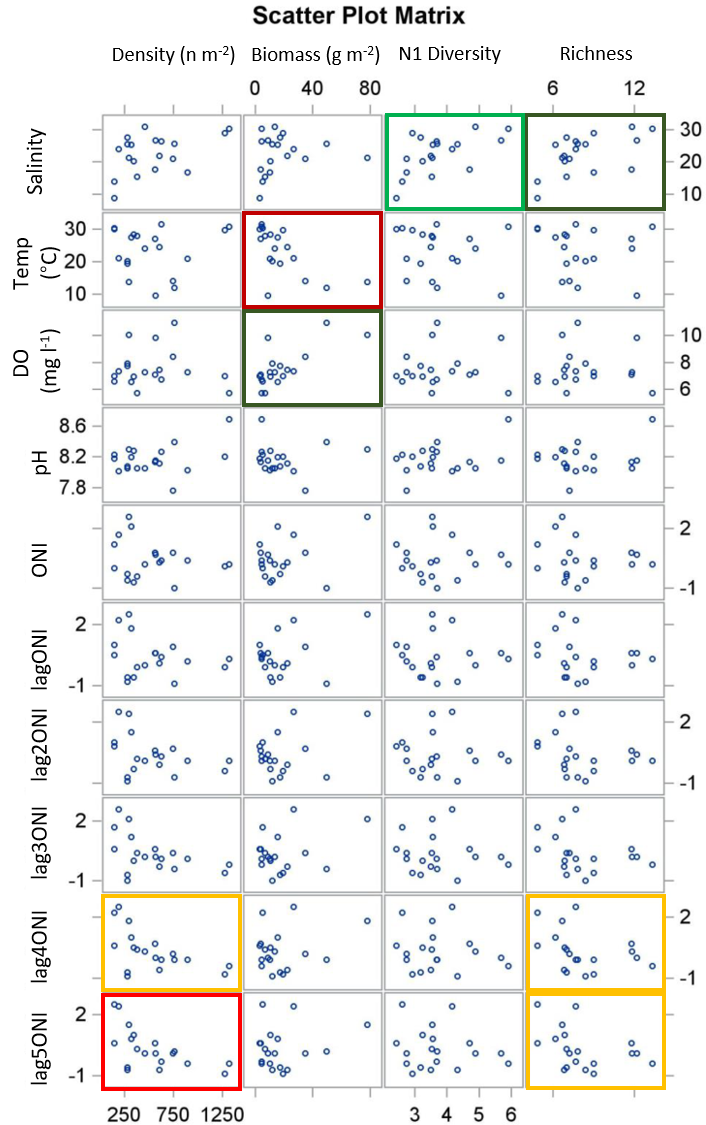

Supplement: S6 Fig — See S4 Table for r and p-values. Plots depicting negative relationships are outlined with dark red (r ≤ -0.60), red (r ≤ -0.50), orange (r ≤ -0.45) and yellow (r ≤ -0.40). Plots depicting positive relationships are outlined with dark green (r ≥ 0.60), medium green (r ≥ 0.50) and light green (r ≥ 0.40). (TIF) [file pone.0255931.s010.tif]

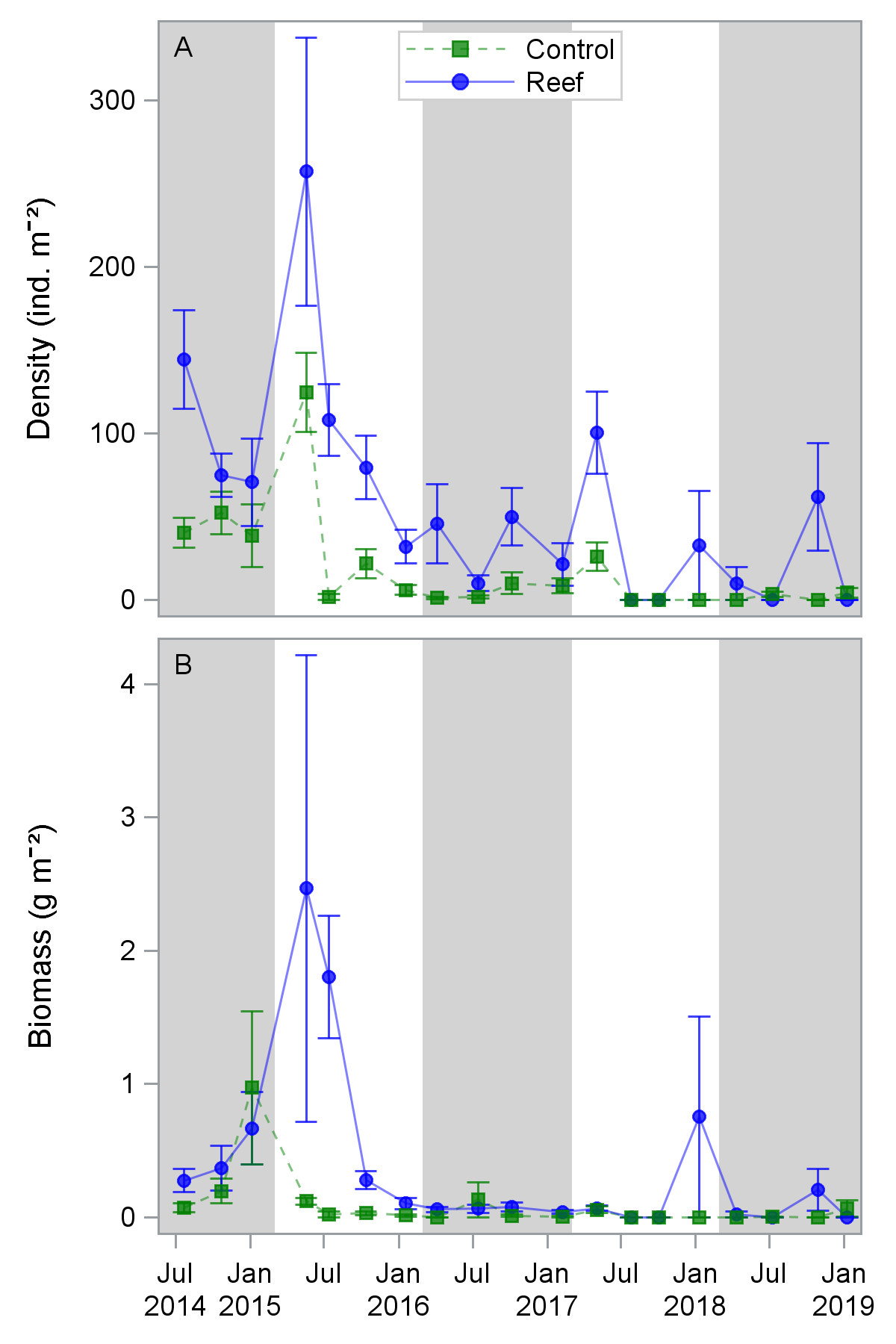

Supplement: S7 Fig — Mean (SE) Xanthidae density (A) and biomass (B), and salinity and ONI (C) measured quarterly from January 2014-January 2019. ONI data are from [18]. (TIF) [file pone.0255931.s011.tif]

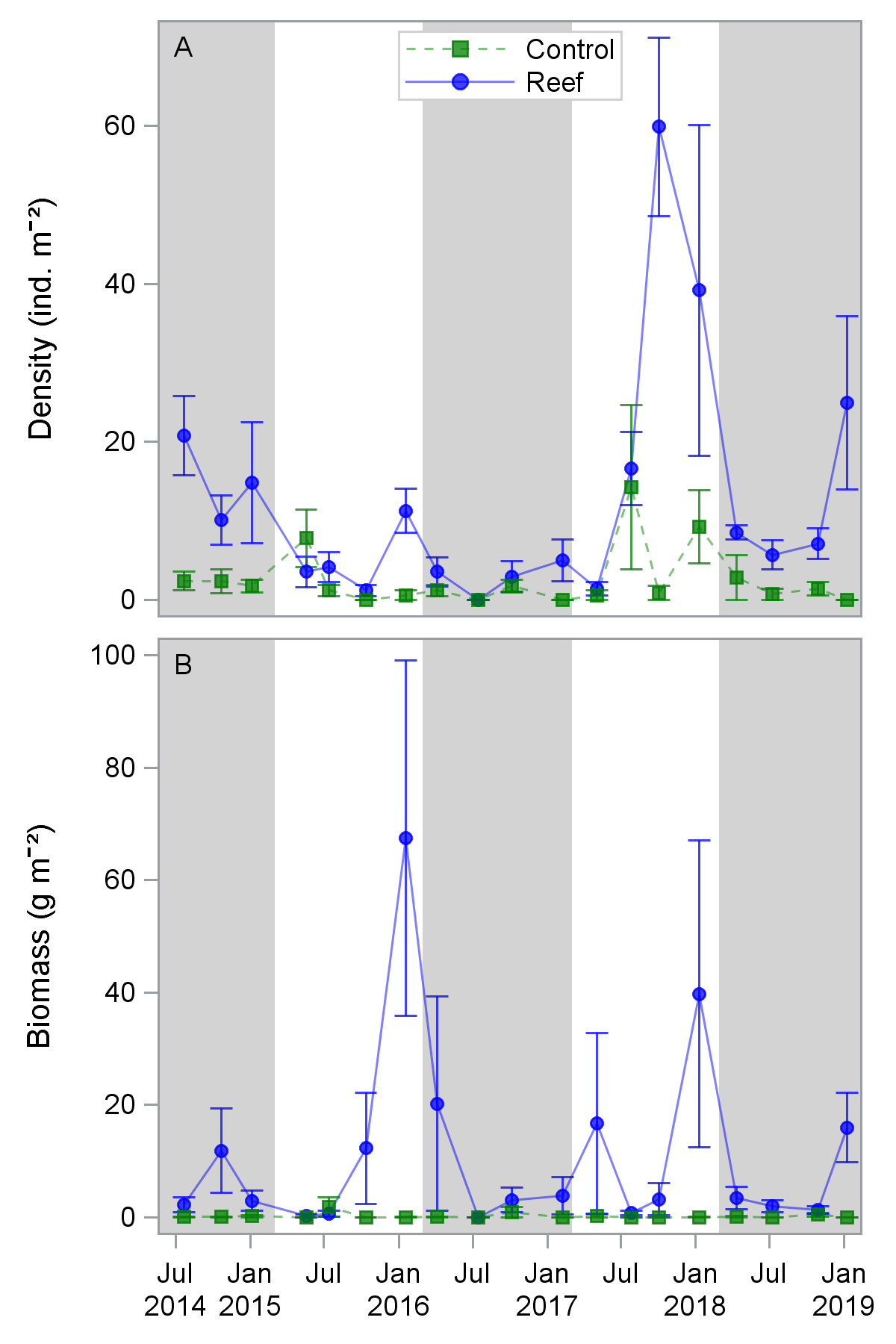

Supplement: S8 Fig — Mean (SE) Menippe adina density (A) and biomass (B), and salinity and ONI (C) measured quarterly from January 2014-January 2019. ONI data are from [18]. (TIF) [file pone.0255931.s012.tif]
